# A Case of Myxoma Mimicking Scapulothoracic Bursa

**DOI:** 10.7759/cureus.44973

**Published:** 2023-09-10

**Authors:** Rabia Bahauddin, Renan Adam

**Affiliations:** 1 Radiology, Hamad Medical Corporation (HMC), Doha, QAT; 2 Musculoskeletal Radiology, Hamad Medical Corporation (HMC), Doha, QAT

**Keywords:** clinical imaging, musculoskeletal imaging, case report, scapulothoracic bursitis, myxomatous tumors

## Abstract

Myxoid soft tissue tumors are an unusually diverse group of neoplasms, most commonly involving the extremities. They are mesenchymal neoplasms characterized by the abundant production of myxoid matrix with a gelatinous appearance. They have varying characteristics in medical imaging and histopathology. The prognosis is variable for recurrence. Less regularly, they are found in bone, skin, the genitourinary tract, aponeurotic tissue, and subcutaneous tissue. We present a case of myxomatous tumor at the scapulothoracic region mimicking bursitis.

## Introduction

Myxoid tumors are a heterogenous group of mesenchyme-rich tumors encompassing everything from entirely benign to non-metastasized malignant and metastasized malignant neoplasms. They show considerable overlap in characteristics on clinical examination and on imaging. They are most commonly seen arising from skeletal muscles in the trunk. Their water content is excessive, which gives them low attenuation on CT, high signal on T2-weighted imaging, and low signal on T1-weighted imaging [1]. Intramuscular myxomas are predominantly found in middle-aged adults, with a female predominance. The tumor is usually solitary, slowly growing, and asymptomatic [2]. A conclusive diagnosis can only be made by histopathological investigation, with surgical excision being the therapy of choice for symptomatic patients.

## Case presentation

**Figure 1:**
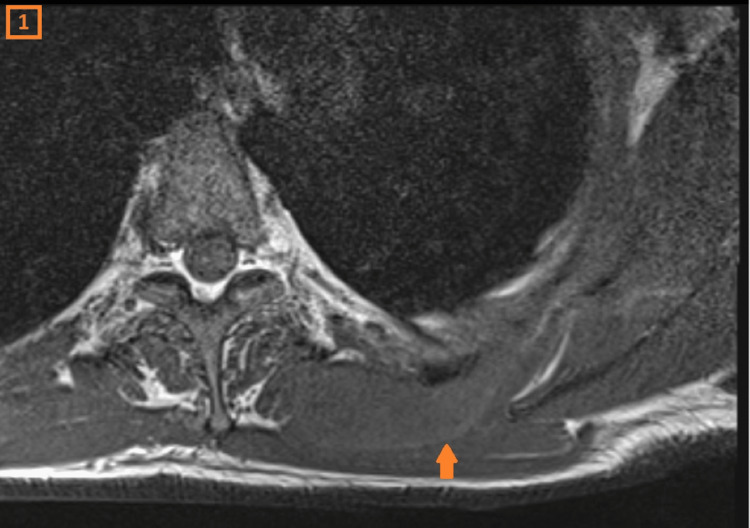
Cystic lesion as seen on axial T1-weighted image

**Figure 2:**
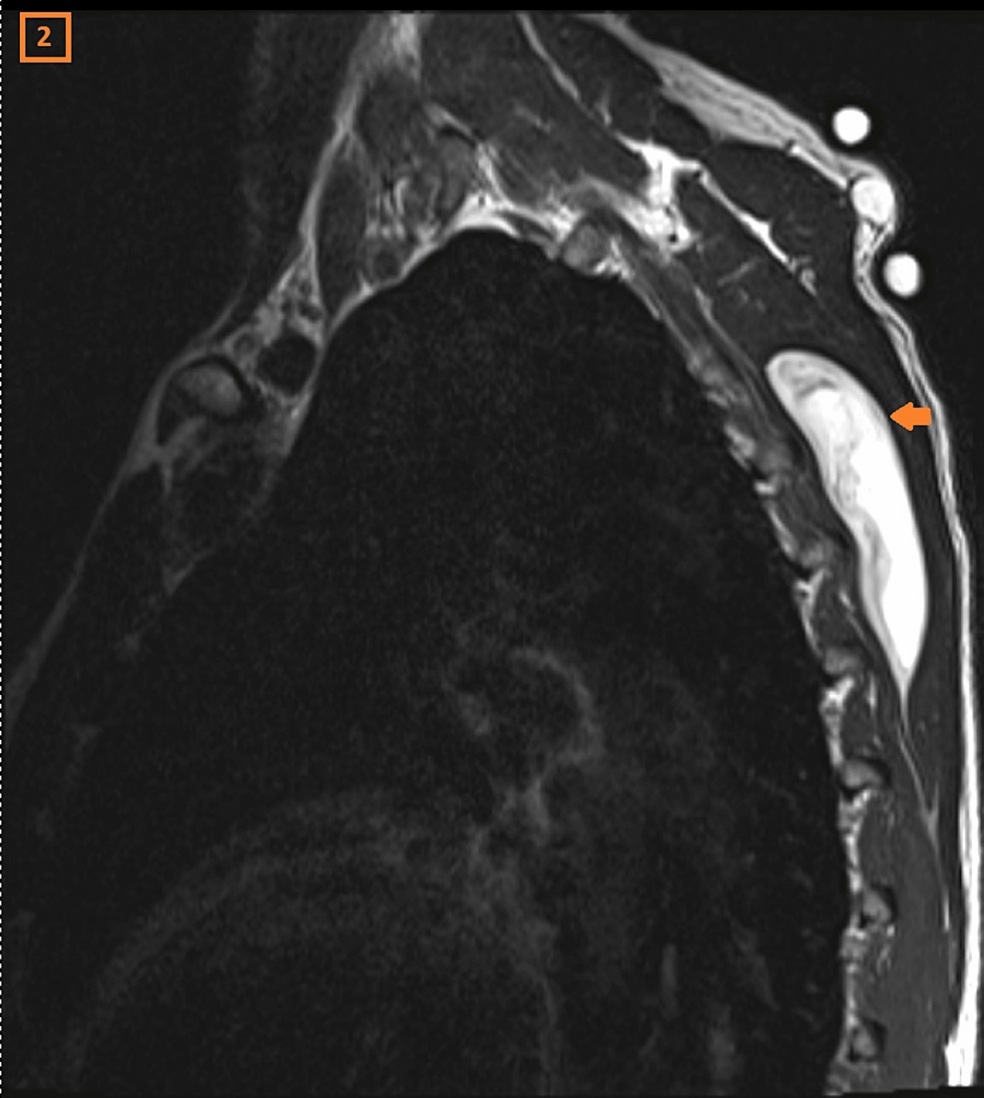
Cystic lesion as seen on sagittal T2-weighted image

**Figure 3:**
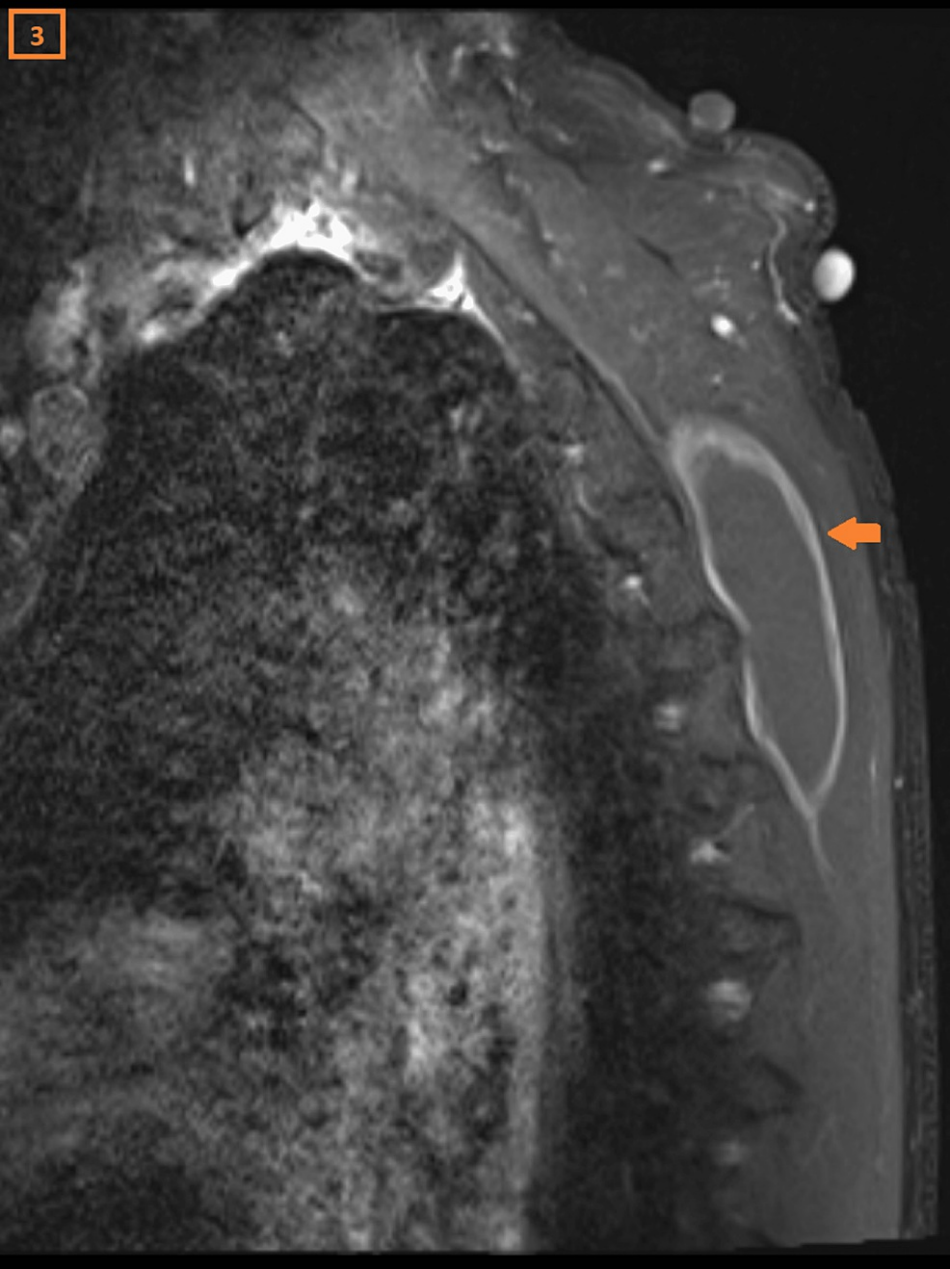
Post-contrast image of cystic lesion The axial T1, sagittal T2, and post-contrast MRI images of the scapulothoracic region show an elongated lesion hyperintense on T2, and isointense on T1 with peripheral contrast enhancement.

A 56-year-old male patient had been experiencing pain for six months in his left shoulder. Clinically, two mass lesions were noted on the back side of the shoulder. The largest mass was mobile, with snapping features noted during scapular movement. The MRI showed a cystic lesion in the scapulothoracic region, measuring 8 cm x 1.5 cm. It demonstrated intermediate signal intensity on T1 (Figure 1), high signal intensity on T2-weighted images (Figure 2), and peripheral contrast enhancement on the post-contrast images (Figure 3).

Considering the location and signal intensity, the features mimicked scapulo-thoracic bursitis. A US-guided biopsy was done, and the histopathology revealed a myxoid neoplasm. The lesion was planned for surgical excision. No acute or chronic complications were noted. There was no recurrence. 

## Discussion

Myxoid tumors are a group of malignant and benign lesions distinguished by myriad extracellular myxoid (mucoid) matrixes. They fluctuate in their clinical behavior, ranging from totally safe lesions to locally aggressive and malignant tumors that frequently metastasize. There is a sizeable overlap radiologically and clinically between numerous myxoid lesions, creating a challenge for interdisciplinary teams [1]. 

The water content of the myxoid matrix is extremely high. Hence, it has a hypoechoic appearance on US, a low signal on T1-weighted images, and a high signal on T2-weighted images, along with a low attenuation on CT imaging. The modality of choice for myxoid tumors is MRI [2]. They are more commonly found intramuscularly and rarely subcutaneously. The most familiar site of occurrence is the thigh. They commonly occur as painless, growing soft tissue masses [3]. 

Differential diagnosis via imaging includes ganglion cysts, nerve sheaths, and other myxoid neoplasms. The well-defined appearance, along with location, perilesional fat or edema, T1/T2 signal intensities, and contrast enhancements, are important in differentiating myxomatous tumors from other lesions [3,4]. On CT imaging, they look like well-circumscribed lesions with low density and mild contrast enhancement. On MRI imaging, they are well-defined with even or mildly lobulated borders. They show irregular, heterogeneous, and occasionally avid contrast enhancement on contrast-enhanced images. Peritumoral rind of fat or edema is suggested to be an extremely likely diagnosis on MRI imaging [3-5]. Immunohistochemical examinations of intramuscular myxomas reveal diffuse positivity for vimentin in the cells, along with focal weak positivity for CD34. It shows negativity for S-100 [6]. 

Scapulothoracic bursitis, on the other hand, is usually seen as a well-circumscribed cystic lesion between the serratus anterior and the thoracic wall [7]. The signal intensity of most of the scapulothoracic bursitis on MRI is low on T1-weighted images and high on T2-weighted images. Post-contrast T1-weighted images showed rim-like enhancement of the cyst wall. These features are typical of the cystic nature of the lesions [7]. 

Since our patient’s mass lesion was in a location that is unusual for myxomatous lesions, the top differential was a cystic lesion, more specifically bursitis. However, a biopsy was done to confirm the diagnosis, which in turn confirmed a myxoid neoplasm. Surgical removal with broad margins is the treatment option of choice with scarce recurrence [8]. 

## Conclusions

Myxomatous tumors are rare in the scapulothoracic region. Therefore, while assessing soft tissue lesions, it should be kept in mind that some lesions that are extremely hyperintense on T2-weighted images can, from time to time, imitate cysts. Imaging, along with a biopsy, is definitively required for the proper diagnosis to differentiate myxomatous tumors from other cystic lesions.
